# Side-by-Side Comparison of uPAR-Targeting Optical Imaging Antibodies and Antibody Fragments for Fluorescence-Guided Surgery of Solid Tumors

**DOI:** 10.1007/s11307-021-01657-2

**Published:** 2021-10-12

**Authors:** Victor M. Baart, Labrinus van Manen, Shadhvi S. Bhairosingh, Floris A. Vuijk, Luisa Iamele, Hugo de Jonge, Claudia Scotti, Massimo Resnati, Robert A. Cordfunke, Peter J. K. Kuppen, Andrew P. Mazar, Jacobus Burggraaf, Alexander L. Vahrmeijer, Cornelis F. M. Sier

**Affiliations:** 1 Department of Surgery, Leiden University Medical Center, Leiden, The Netherlands; 2 Unit of Immunology and General Pathology, Department of Molecular Medicine, University of Pavia, Pavia, Italy; 3 Ardis Srl, Pavia, Italy; 4 Age Related Diseases Unit, Division of Genetics and Cell Biology, San Raffaele Scientific Institute, Milano, Italy; 5 Department of Immunohematology and Blood Transfusion, Leiden University Medical Center, Leiden, The Netherlands; 6 Monopar Therapeutics, Wilmette, IL USA; 7 Centre for Human Drug Research, Leiden, The Netherlands; 8 Percuros BV, Leiden, The Netherlands

**Keywords:** Image-guided surgery, Near infrared, Fluorescence, Urokinase plasminogen activator receptor, uPAR, IgG, F(ab’)2, Fab, Pancreatic cancer, Head and neck cancer, Oral cancer, Colon cancer, Fluorescence-guided surgery

## Abstract

**Purpose:**

Radical resection is paramount for curative oncological surgery. Fluorescence-guided surgery (FGS) aids in intraoperative identification of tumor-positive resection margins. This study aims to assess the feasibility of urokinase plasminogen activator receptor (uPAR) targeting antibody fragments for FGS in a direct comparison with their parent IgG in various relevant in vivo models.

**Procedures:**

Humanized anti-uPAR monoclonal antibody MNPR-101 (uIgG) was proteolytically digested into F(ab’)2 and Fab fragments named uFab2 and uFab. Surface plasmon resonance (SPR) and cell assays were used to determine *in vitro* binding before and after fluorescent labeling with IRDye800CW. Mice bearing subcutaneous HT-29 human colonic cancer cells were imaged serially for up to 120 h after fluorescent tracer administration. Imaging characteristics and ex vivo organ biodistribution were further compared in orthotopic pancreatic ductal adenocarcinoma (BxPc-3-luc2), head-and-neck squamous cell carcinoma (OSC-19-luc2-GFP), and peritoneal carcinomatosis (HT29-luc2) models using the clinical Artemis fluorescence imaging system.

**Results:**

Unconjugated and conjugated uIgG, uFab2, and uFab specifically recognized uPAR in the nanomolar range as determined by SPR and cell assays. Subcutaneous tumors were clearly identifiable with tumor-to-background ratios (TBRs) > 2 after 72 h for uIgG-800F and 24 h for uFab2-800F and uFab-800F. For the latter two, mean fluorescence intensities (MFIs) dipped below predetermined threshold after 72 h and 36 h, respectively. Tumors were easily identified in the orthotopic models with uIgG-800F consistently having the highest MFIs and uFab2-800F and uFab-800F having similar values. In biodistribution studies, kidney and liver fluorescence approached tumor fluorescence after uIgG-800F administration and surpassed tumor fluorescence after uFab2-800F or uFab-800F administration, resulting in interference in the abdominal orthotopic mouse models.

**Conclusions:**

In a side-by-side comparison, FGS with uPAR-targeting antibody fragments compared with the parent IgG resulted in earlier tumor visualization at the expense of peak fluorescence intensity.

**Supplementary Information:**

The online version contains supplementary material available at 10.1007/s11307-021-01657-2.

## Introduction

Cancer incidence is on the rise with one in five to six people awaiting a cancer diagnosis and one in eight to ten people eventually succumbing to the disease [1]. Despite recent advances in treatment, surgical resection remains the corner stone of curative therapy [1, 2]. The primary aim of oncological surgery is to achieve local control by radical resection (i.e., R0, tumor-negative margins), as tumor-positive margins negatively influence disease-free and overall survival [3–5]. As a result, correctly discerning malignant from benign tissue during surgery is fundamental for resection.

Surgeons can utilize intraoperative imaging techniques to aid in this differentiation. A relatively novel technology, fluorescence-guided surgery (FGS), uses advanced camera systems to capture near-infrared (NIR) fluorescence emitted by contrast agents, targeting, for example, ureters, nerves, or tumors. FGS has the advantage that it is real-time, has a high contrast and sensitivity, does not utilize ionizing radiation, and is easy-to-use [6, 7]. For oncological FGS, the crux lies in developing fluorescent tracers that specifically target proteins which are present in malignant cells and absent or quiet in surrounding tissue. A promising target for imaging of disease, including various types of cancer, is the urokinase plasminogen activator receptor (uPAR) [8, 9].

uPAR is a three-domain cell membrane bound receptor that plays a pivotal role in growth factor activation and extracellular matrix remodeling, stimulating proliferation, differentiation, and migration of cells. Its presence in cancer correlates with prognostic outcome variables such as metastatic disease and reduced overall- and disease-free survival. Furthermore, immunohistochemical studies have revealed absent to low levels of uPAR expression in healthy homeostatic tissue whereas at the interface between tumor and healthy tissue, uPAR is highly overexpressed on both tumor and tumor-associated stromal cells [8–12]. Such a pattern is ideal for imaging and, not surprisingly, a PET tracer is currently undergoing extensive clinical trials in order to image “cancer aggressiveness” and subsequently aid in tailoring treatment options to disease biology [13].

As uPAR expression is highest at the tumor borders, our group has primarily focused on developing uPAR-targeted FGS tracers. We have successfully targeted uPAR for FGS in various *in vivo* human cancer models using a mouse monoclonal antibody and more recently with MNPR-101, a first-in-class humanized monoclonal antibody targeting domain three of uPAR [14–16]. This domain is ideal as recognition is independent of the often simultaneously upregulated uPAR-ligands urokinase and vitronectin, and remains membrane bound when uPAR is cleaved [9].

The relatively large size of antibodies (≈ 150 kiloDalton (kDa)), however, might limit extravasation, and the interaction of their crystallizable fragment (Fc) with the neonatal Fc receptor on endothelial cells results in an extended half-life, decreasing tissue penetration and delaying the optimal imaging window [17, 18]. As a consequence, there is often a delay of 4 to 6 days after administration before patients can be imaged with optimal results [19]. Reducing molecular size, the dominant variable in determining extravasation (distribution) and clearance, shortens imaging times and improves tumor penetration [20]. Clinically, these advantages should result in improved tumor fluorescence and contrast at earlier time points resulting in better visualization for surgeons and more rapid surgery, also allowing surgeons to utilize fluorescence in (semi-) acute settings [21]. As a result, shortening the imaging window is often presented as the holy grail of FGS when introducing novel small-sized fluorescent tracers. Tumor specificity, tumor-to-background ratio (TBR), and tumor mean fluorescence intensity (MFI), however, are of equal importance. Positron emission tomography (PET) studies have for instance illustrated that antigen binding fragments (Fab) and F(ab’)_2_ lead to much faster tumor targeting, but also a nearly two- to three-fold lower peak uptake compared to the parent antibody [22, 23]. Whether this is also true for FGS remains to be elucidated. The current study aims to determine how uPAR-targeting Fab and F(ab’)_2_ compare to their parent full-sized antibody (MNPR-101) in FGS of orthotopic PDAC, HNSSC, and peritoneal carcinomatosis CRC models.

## Materials and Methods

### Generation of Fab and F(ab’)_2_ from MNPR-101

MNPR-101, a humanized antibody targeting uPAR, was enzymatically fragmented into Fab and F(ab’)_2_ by respectively GingisKHAN and FabRICATOR kits, following the protocols of the manufacturer (Genovis AB, Lund, Sweden). The full-sized antibody and the generated fragments are further indicated as uIgG, uFab2, and uFab. Tracers (full-sized antibody and fragments) were conjugated with IRDye 800CW-NHS ester (from here on 800F; LI-COR biotechnology, Lincoln, USA) according to the manufacturer and as published before [16]. Digestion and conjugation results were evaluated using SDS-PAGE on pre-casted 4–20% gels (Criterion, Bio-Rad laboratories, Veenendaal, The Netherlands). Proteins on gels were stained with Coomassie brilliant blue G-250 (Bio-Rad laboratories, Veenendaal, The Netherlands) and fluorescence of 800F was determined using an Odyssey imager (LI-COR biotechnology, Lincoln, USA). Degrees of labeling were calculated and verified by MALDI-TOF mass spectrometry as described previously [24].

### Surface Plasmon Resonance of Antibody Fragments

All surface plasmon resonance (SPR) experiments were performed on a Biacore T200 (GE Healthcare Life Sciences, Uppsala, Sweden) using a NiHC1500M sensor chip (Xantec Bioanalytics, Düsseldorf, Germany) as described previously (see ESM for details) [16].

### Cells and Culture Conditions

Culture conditions of human embryonic kidney empty vector (HEK EV), HEK uPAR wildtype (HEK uPAR WT), HEK uPAR D2-D3 isoform (HEK uPAR D2-3), OSC-19-luc2-GFP, BxPC-3-luc2, and HT-29-luc2 cells are described in the ESM file.

### Flow Cytometry and Cell-Based Plate Assays

For flow cytometry, cells were incubated in succession with primary antibody (-fragment), secondary anti-human IgG Fab fragment antibody (clone 4A11; ab771, Abcam, Cambridge, UK), and anti-mouse 488 antibody (A21121, Life Technologies, CA, USA) using a standard flow cytometry protocol and measured on a LSRII flow cytometer (BD Biosciences, CA, USA). Binding of serially diluted 800F-labeled antibodies to cells cultured in 96-well plates was determined using the Odyssey and fluorescence was corrected for cell density with a ToPro-3 iodide nuclear staining (T3605, Invitrogen, CA, USA). The CD52-targeting humanized antibody Alemtuzumab coupled to 800F functioned as a conjugate control. For detailed methods, see ESM.

### *In Vivo *Tumor Models

All *in vivo* experiments were approved by the Dutch Central Commission for Animal Experimentation (AVD1160020172925) and performed in accordance with the code of practice “Dierproeven In Het Kankeronderzoek”. Six- to ten-week-old female BALB/c-Nude mice (CAnN.Cg-*Foxn1nu*/Crl, Charles River Laboratories, MA, USA) were inoculated with tumor cells and randomized into experimental groups once tumors had reached predetermined sizes by digital caliper or bioluminescence using the IVIS® Spectrum Preclinical *In Vivo* Imaging System (Spectrum, PerkinElmer, MA, USA) (for details, see ESM). Subcutaneous tumors were induced by subcutaneous injection of 500,000 HT-29 cells. Orthotopic OSC-19-luc2-GFP HNSCC, BxPC-3-luc2 PDAC, and HT-29-luc2 CRC peritoneal carcinomatosis models were induced as previously published [15, 25, 26].

### Fluorescence Imaging Systems

Fluorescence images were acquired using the preclinical Pearl Trilogy Small Animal Imaging System (Pearl; LI-COR biotechnology, Lincoln, USA) and the Artemis clinical system (Quest Medical Imaging, Middenmeer, The Netherlands). Pearl images were analyzed with Image Studio 5.2 (LI-COR biotechnology, Lincoln, USA). Frames from Artemis recordings, taken with standardized exposure times, were captured with Spectrum Capture Suite 1.4.3 (Quest Medical Imaging, Middenmeer, The Netherlands) and analyzed with Fiji Image-J [27].

### Fluorescence Imaging

One hundred microliters of fluorescent tracer was administered intravenously at equal number of antigen binding sites, respectively, 1 nmol for uIgG-800F and uFab2-800F and 2 nmol for uFab-800F. Subcutaneous models were imaged at 1, 2, 4, 12, 24, 36, 48, 72, 96, and 120 h while orthotopic models were imaged at the optimal imaging window after tumor exposure. Optimal time points were selected based on TBR and MFI cut-offs, literature, and visual interpretation. Outcome measures included TBR, MFI, and Artemis exposure times (see ESM for details).

### Post-mortem Histological Analysis

Tumors were resected with surrounding normal tissue, embedded in paraffin, sectioned, and scanned for 800-nm fluorescence using the Odyssey. After scanning, sections were stained for hematoxylin and eosin and digitalized with the Panoramic Digital slide Scanner and CaseViewer 2.3 (both 3D Histech, Budapest, Hungary). Overlays were created with Adobe Photoshop CC 2018 (Adobe Systems, CA, USA).

### Image Analysis and Statistics

TBRs were calculated by dividing the tumor MFI with background MFI measured using the Pearl. For subcutaneous models, the background region-of-interest was the area next to the tumor equidistant from metabolizing organs. For the SSC, PDAC, and CRC peritoneal carcinomatosis models, the background region-of-interests were muscle, spleen, and peritoneum, respectively. Means were reported with standard deviations and compared using two-way ANOVA with GraphPad Prism 8 (GraphPad Software, CA, USA). A *p* value < 0.05 was considered statistically significant.

## Results

### *In Vitro *Characterization of uIgG, uFab2, and uFab

**Fig. 1:**
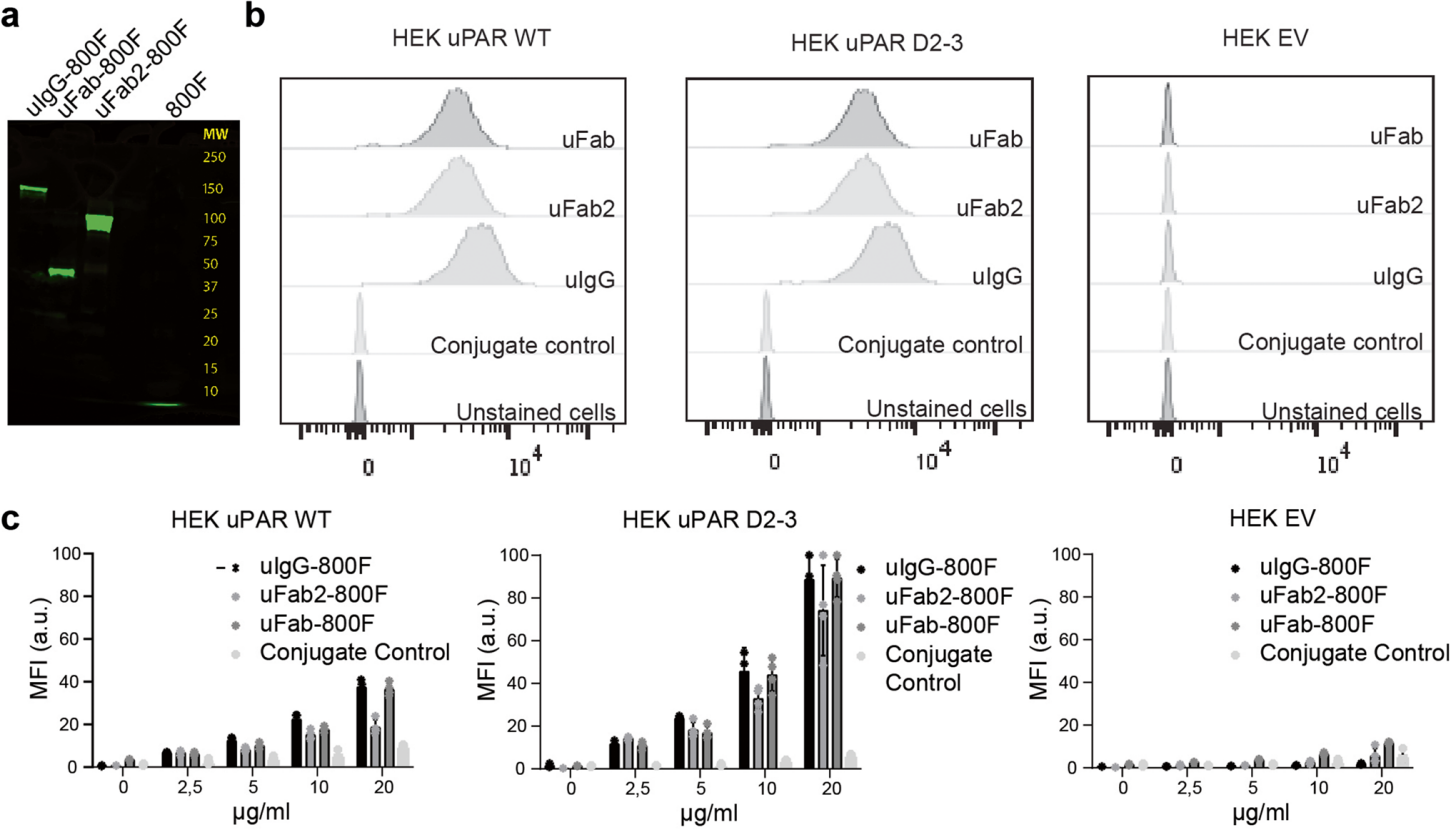
*In vitro* validation of proteolytic cleavage and fluorescent conjugation of uIgG, uFab2, and uFab: **a** fluorescent signal on SDS-PAGE gel at respectively 150–155 kDa, 100–110 kDa, 50–55 kDa, and < 10 kDa for uIgG-800F, uFab2-800F, uFab-800F, and 800F. The latter represents free dye, indicating the purity of the conjugations in the other lanes. **b** Flow cytometry histograms demonstrating retained binding of uFab2 and uFab to wildtype uPAR (left) and the D2-D3 isotype (middle) after proteolytic cleavage of uIgG into the antibody fragments. Specificity is furthermore confirmed using empty vector transfected HEK cells (right) and a conjugate control as controls. **c** Cell-based plate assays showing dose-dependent binding of uIgG-800F, uFab2-800F, and uFab-800F and conjugate control to uPAR-positive HEK WT cells (left), uPAR D2-3 isotype-positive HEK cells (middle), and uPAR-negative EV cells (right). a.u., arbitrary units; MFI, mean fluorescent intensity.

MNPR-101 (150–155 kDa) was enzymatically digested and purified resulting in a 100–110 kDa uFab2-800F and 50–55 kDa uFab (Fig. 1a). SPR determined the K_D_ of uIgG, uFab2, and uFab for recombinant uPAR to be 2.19 × 10^−10^ ± 2.41 × 10^−11^ M, 5.61 × 10^−10^ ± 1.61 × 10^−11^ M, and 8.66 × 10^−10^ ± 4.17 × 10^−10^ M, respectively (Table 1; for sensograms, see ESM file, Suppl. Figure 1). Affinities did not differ significantly from each other (*p* = 0.59). *K*_a_ and *K*_d_ values of all tracers can be found in Suppl. Table 1 (see ESM). Flow cytometry confirmed specificity for uPAR by showing a right shift in signal when uIgG, uFab2, and uFab were incubated with HEK uPAR WT cells, HEK uPAR D2-3 cells, but not with HEK EV cells (Fig. 1b).

**Table 1:** Comparison of uIgG, uFab2, and uFab characteristics

Tracer	Targeting domain (Aa uPAR)	MW (kDa)	Valency	K_D_ (M)	Label	Degree of labeling	K_D_ after conjugation (M)	Pre-/post-labeling K_D_ difference (*T* test; *p*)
uIgG	268–275	150–155	2	2.19 × 10^−10^ ± 2.41^−11^	800F	0.9	2.56 × 10^−10^ ± 4.71^−11^	0.47
uFab2	268–275	100–110	2	5.61 × 10^−10^ ± 1.61^−10^	800F	0.9	3.84 × 10^−10^ ± 1.51^−10^	0.53
uFab	268–275	50–55	1	8.66 × 10^−10^ ± 4.17^−10^	800F	1.2	1.43 × 10^−9^ ± 2.66^−10^	0.32

The calculated fluorescence labeling with 800F was comparable for uIgG, uFab2, and uFab, and the *K*_D_ did not differ significantly after labeling (uIgG vs. uIgG-800F *p* = 0.47; uFab2 vs. uFab2-800F *p* = 0.53; uFab vs. uFab-800F *p* = 0.32; Table 1). Cell-based plate assays showed a dose-dependent increase of the MFI on HEK uPAR WT cells and the HEK uPAR D2-3 cells, but not on HEK EV control cells (Fig. 1c).

### Serial Imaging of Subcutaneous HT-29 Tumors with uIgG-800F, uFab2-800F, and uFab-800F

**Fig. 2:**
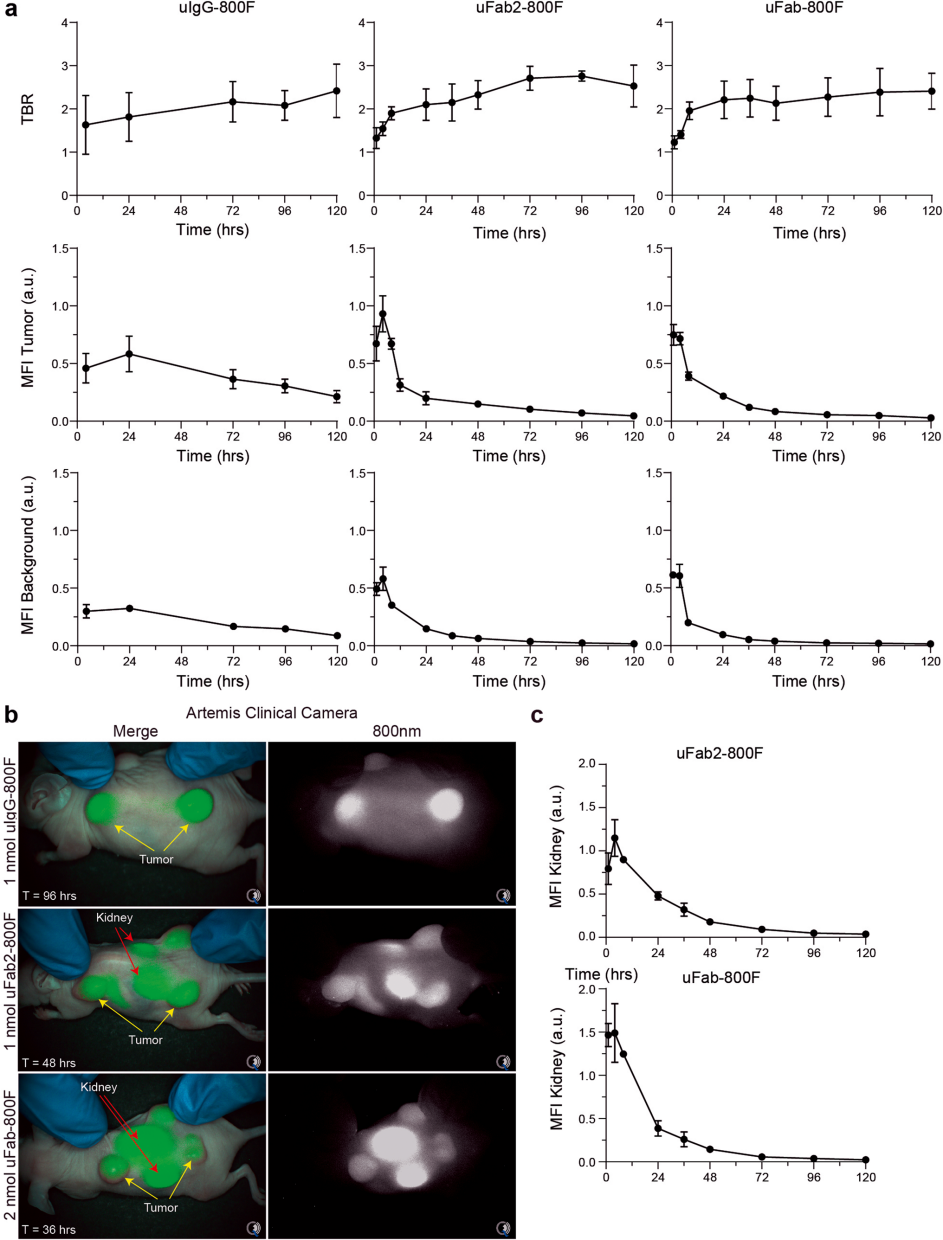
*In vivo* serial imaging of uIgG-800F, uFab2-800F, and uFab-800F in subcutaneous HT-29 tumor bearing mice: **a** TBR and MFI measured using the Pearl over time after injection of 1-nmol uIgG-800F, 1-nmol uFab2-800F, and 2-nmol uFab-800F. **b** Merge and 800-nm images taken with the Artemis clinical camera demonstrating real-time tumor imaging at 96 h for uIgG-800F, 48 h for uFab2-800F, and 36 h for uFab-800F. These time slots are shown as they fall within the optimal imaging window for each of the tracers. For images at other time periods, see Suppl. Figure 2 (ESM). Tumor and kidney fluorescence are identified with yellow and red arrows, respectively. **c** Transcutaneous kidney fluorescence over time after uFab2-800F and uFab-800F administration. a.u., arbitrary units; hrs, hours; MFI, mean fluorescence intensity; TBR, tumor-to-background ratio; T, time.

Imaging of subcutaneous HT-29 tumors in mice demonstrated tumor specificity and identified the imaging window of 1-nmol uIgG-800F, 1-nmol uFab2-800F, and 2-nmol uFab-800F. Tumor accumulation of the full-sized antibody uIgG-800F could be seen as early as 1 h after administration, but TBRs did not surpass two, an arbitrary cut-off point deemed suitable for NIR imaging, until 72 h, due to relatively high background fluorescence at earlier time points [28]. The antibody fragments uFab2-800F and uFab-800F accumulated more rapidly in tumors, with TBRs surpassing two after 24 h (Fig. 2a; for serial images, see Suppl. Figure 2, see ESM).

Tumor fluorescence consistently remained above 0.1 arbitrary units (a.u.) with uIgG-800F, but dipped below it after 72 h with uFab2-800F and 36 h with Fab-800F (MFI uIgG-800F 120 h = 0.156 ± 0.016 au; MFI uFab2-800F 72 h = 0.103 ± 0.017 au; MFI uFab-800F 36 h = 0.213 ± 0.050 au; Fig. 2a). During the imaging windows 72–120 h, 24–72 h, and 24–36 h, subcutaneous tumors were clearly identifiable with the clinical Artemis camera after uIgG-800F, uFab2-800F, or uFab-800F administration, respectively (Fig. 2b). Extra-tumoral uIgG-800F accumulation was not visible. At all time points, kidney fluorescence could be seen through the skin after uFAb2-800F and uFab-800F injection with kidney fluorescence surpassing tumor fluorescence (Fig. 2b–c).

### Fluorescence Imaging of Orthotopic Tumor Models with uIgG-800F, uFab2-800F, and uFab-800F

Using three orthotopic cancer models, BxPC-3 PDAC, OSC-19 tongue SCC, and HT-29 CRC peritoneal carcinomatosis, FGS potential of uIgG-800F, uFab2-800F, and uFab-800F was compared. MFI of 1-nmol uIgG-800F, 1-nmol uFab2-800F, and 2-nmol uFab-800F in 100 µL solution was 2.08 ± 0.38, 2.47 ± 0.44, and 4.1 ± 0.77 au, respectively. Tumor burdens, measured by bioluminescence, did not differ significantly between tracers at the time of injection (*p* = 0.72 for BxPC-3-luc2; *p* = 0.72 for OSC-19-luc2-GFP; *p* = 0.31 for HT29-luc2; Suppl. Figure 3, see ESM). Tumors were imaged at 96 h for uIgG-800F, 48 h for uFab2-800F, and 36 h for Fab-800F after tracer administration.

**Fig. 3:**
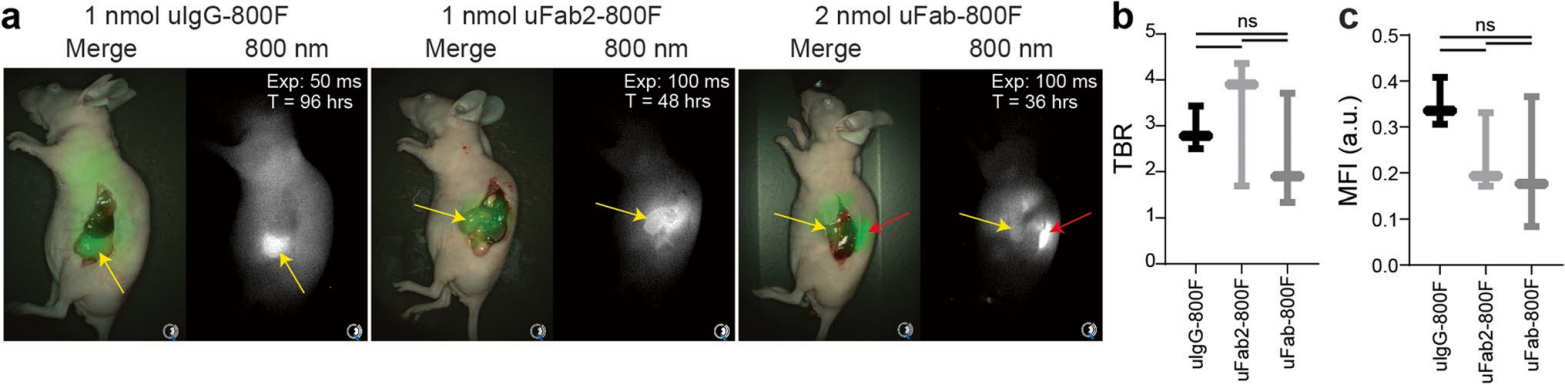
*In vivo* fluorescence imaging of orthotopic BxPC-3 pancreas adenocarcinomas with uIgG-800F, uFab2-800F, and uFab-800F during the optimal time period: **a** NIR images of orthotopic BxPC-3 pancreas adenocarcinomas taken with the Artemis clinical camera 96 h are uIgG-800F (left panel), 48 h are uFab2-800F (middle panel), and 36 h after uFab-800F (left panel) administration. Tumors are identified with a yellow arrow and kidney fluorescence, when present, with a red arrow. Note the lower exposure time needed after uIgG-800F administration reflecting a higher MFI. **b** TBRs and **c** tumor MFIs measured using the Pearl do not differ significantly between uIgG-800F, uFab2-800F, and uFab-800F at respectively 96 h, 48 h,and 36 h after injection. a.u., arbitrary units; hrs, hours; MFI, mean fluorescence intensity; ns, not significant; TBR, tumor-to-background ratio; T, time.

Fluorescence clearly accumulated in abdominal PDAC BxPC-3-luc2 tumors after intravenous injection of the various tracers. In particular with uFab-800F, transcutaneous kidney and liver fluorescence interfered with optimal tumor imaging (Fig. 3a). TBRs were 2.5 ± 0.4 for uIgG-800F, 3.3 ± 1.2 for uFab2-800F, and 2.3 ± 1.0 for uFab-800F. TBRs did not differ between all 3 tracers (*p* = 0.58; Fig. 3b). Tumor MFI did not differ significantly but tumors could be visualized with shorter exposure times after uIgG-800F injection as opposed to uFab2-800F or uFab-800F (*p* = 0.32; multiple comparisons, uIgG-800F vs. uFab2-800F *p* = 0.32, uIgG-800F vs. uFab-800F *p* = 0.46, uFab2-800F vs. uFab-800F *p* = 0.95; Fig. 3c).

**Fig. 4:**
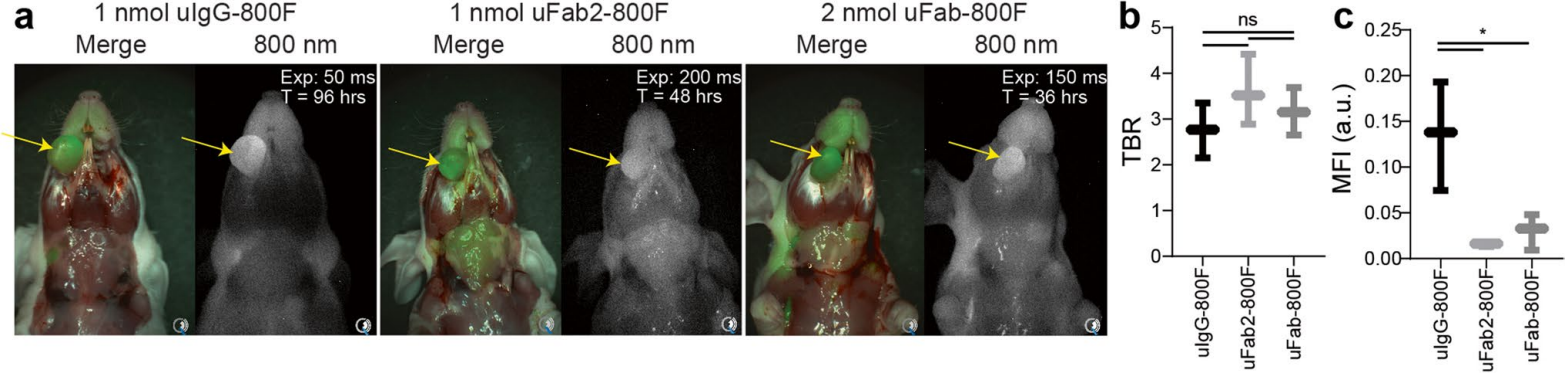
*In vivo* fluorescence imaging of uIgG-800F, uFab2-800F, and uFab-800F during the optimal imaging window in orthotopic squamous cell OSC-19 tongue tumors: **a** NIR images taken with the Artemis clinical camera of orthotopic OSC-19 tongue tumors 96 h after uIgG-800F, 48 h after uFab2-800F, and 36 h after uFab-800F administration. Tumors are identified with a yellow arrow. Note the lower exposure time needed to visualize the tumor after uIgG-800F administration compared to uFab2-800F and uFab-800F. **b** TBRs and **c** tumor MFIs measured using the Pearl for uIgG-800F, uFab2-800F, and uFab-800F at respectively 96, 48, and 36 h after administration demonstrating similar TBRs between the groups but a higher MFI for uIgG-800F. a.u., arbitrary units; hrs, hours; MFI, mean fluorescence intensity; NIR, near-infrared; ns, not significant; TBR, tumor-to-background ratio; T, time.

After intravenous injection of uIgG-800F, uFab2-800F, or uFab-800F OSC-19-luc2-GFP, superficial squamous tongue tumors were easily identified with the Artemis clinical system. Shorter exposure times were needed after injection of uIgG-800F as opposed to uFab2-800F or uFab-800F (Fig. 4a). TBRs of 2.8 ± 0.5, 3.6 ± 0.6 and 3.2 ± 0.4 were achieved for uIgG-800F, uFab2-800F, and uFab-800F, respectively. TBRs did not differ significantly between tracers (*p* = 0.33; Fig. 4b). MFI, however, did differ significantly with uIgG-800F having superior absolute fluorescence (*p* = 0.01; multiple comparisons, uIgG-800F vs. uFab2-800F *p* = 0.02, uIgG-800F vs. uFab-800F *p* = 0.03, uFab2-800F vs. uFab-800F *p* = 0.88; Fig. 4c).

**Fig. 5:**
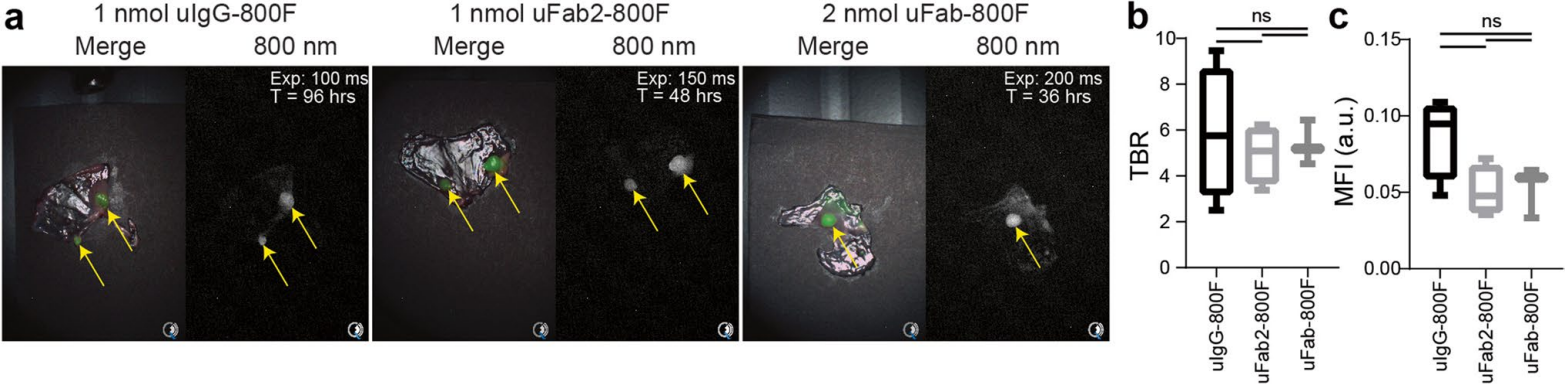
*In vivo* fluorescence imaging of small lesions with uIgG-800F, uFab2-800F, and uFab-800F at the optimal imaging window using an orthotopic HT-29 colorectal cancer peritoneal carcinomatosis model: **a** NIR images taken with the Artemis clinical camera of the peritoneum containing HT-29 lesions 96 h are uIgG-800F, 48 h after uFab2-800F, and 36 h after uFab-800F administration. Tumors are identified with the yellow arrow. Note the differing exposure times needed to create similar images reflecting the differing MFI achieved with each of the tracers. **b** Average TBR and **c** lesion MFI measured using the Pearl achieved 96 h after uIgG-800F, 48 h after uFab2-800F, and 36 h after uFab-800F injection. a.u., arbitrary units; hrs, hours; MFI, mean fluorescence intensity; NIR, near-infrared; ns, not significant; TBR, tumor-to-background ratio; T, time.

Fluorescence detection of small lesions was studied using a HT-29 CRC peritoneal carcinomatosis model. Average lesion diameters were 3.2 ± 0.8 mm, 3.7 ± 1.2 mm, and 4.7 ± 0.6 mm for uIgG-800F, uFab2-800F, or uFab-800F (*p* = 0.18). Similar to the OSC-19-luc2-GFP tongue tumors and the BxPc-3-luc2 PDAC tumors, FGS with the Artemis clinical system was performed with lower exposure times in the uIgG-800F groups compared to the other two groups (Fig. 5a). TBRs, after uIgG-800F, uFab2-800F, or uFab-800F administration, were 5.8 ± 2.5, 4.9 ± 1.1, and 5.4 ± 0.8, respectively, and did not differ significantly between tracer groups (*p* = 0.81; Fig. 5b). Lesion MFI approached statistically significant difference with IgG-800F being superior (*p* = 0.07; multiple comparisons, uIgG-800F vs. uFab2-800F *p* = 0.09, uIgG-800F vs. uFab-800F *p* = 0.14, uFab2-800F vs. uFab-800F *p* = 0.99; Fig. 5c).

### *Ex Vivo *Characterization of Normal and Tumor Tissue

**Fig. 6:**
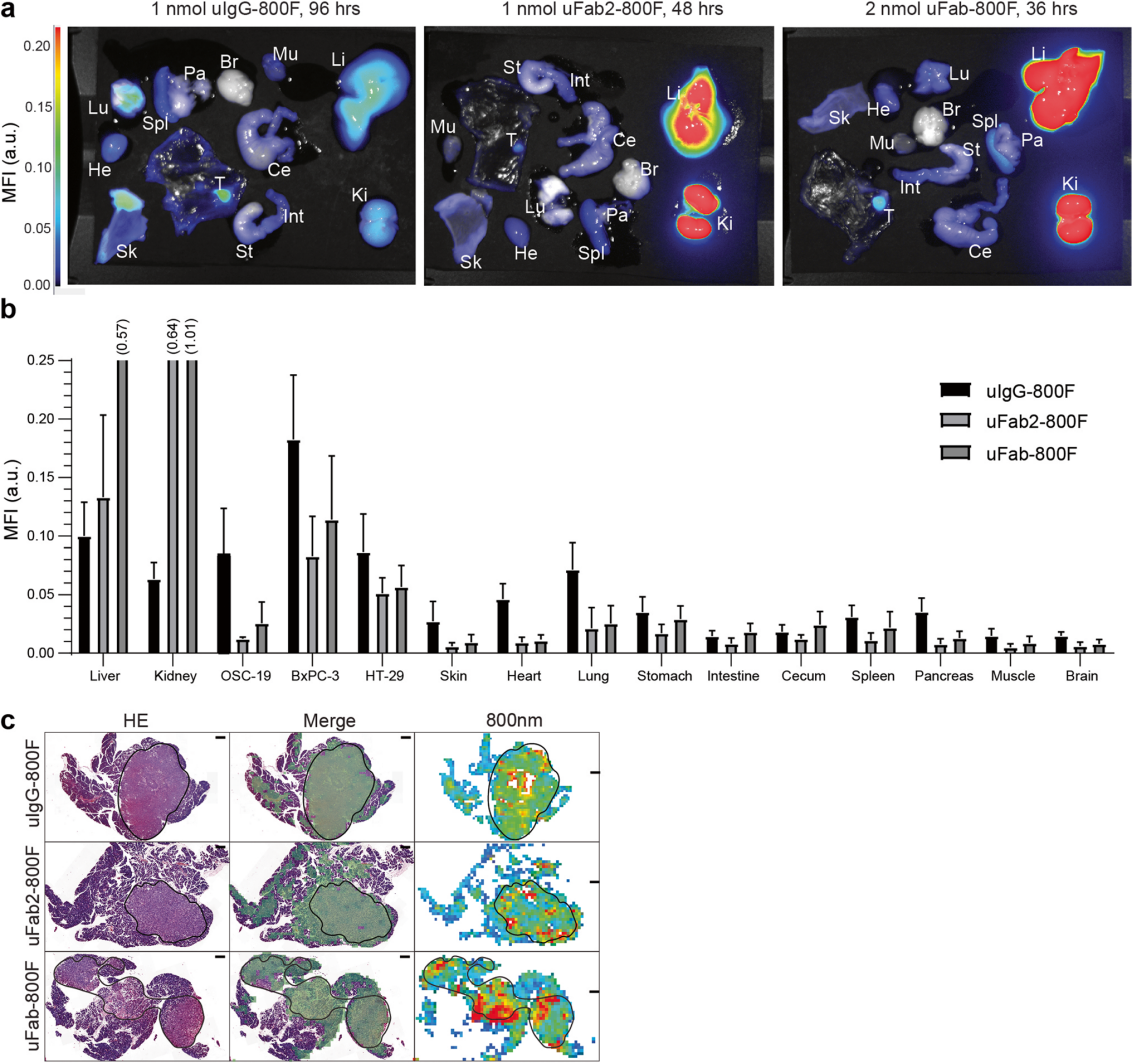
Ex vivo macroscopic and microscopic fluorescence biodistribution of uIgG-800F, uFab2-800F, and uFab-800F at optimal imaging window: **a** ex vivo biodistribution visualized with the Pearl and **b** quantified in a bar graph of uIgG-800F, uFab2-800F, and Fab-800F at respectively 96, 48, and 36 h after administration. Fluorescence intensities across the images are matched. (**c**) Overlay of HE staining and 800-nm fluorescence showing fluorescence accumulation in tumor area compared to surrounding normal tissue. Tumor tissue is delineated by the black line. The black line represents 500 µm. a.u., arbitrary units; Br, brain; Ce, cecum; He, heart; HE, hematoxylin and eosin; Hrs, hours; Int, intestine; Ki, kidneys; Li, liver; Lu, lung; MFI, mean fluorescence intensity; Mu, muscle; nm, nanometer; Pa, pancreas; Sk, skin; Spl, spleen; St, stomach.

Ex vivo macroscopic analysis of normal tissue showed that most of the fluorescence was located in the metabolizing organs (Fig. 6a–b). Liver MFI differed significantly between tracers with uFab-800F having the highest, and uIgG-800F and uFab2-800F having similar MFI’s (*p* < 0.01; multiple comparisons, uIgG-800F vs. uFab2-800F *p* = 0.28, uIgG-800F vs. uFab-800F* p* < 0.01, uFab2-800F vs. uFab-800F *p* < 0.01). Kidney MFI differed significantly between all tracer types with uIgG-800F having the lowest MFI (*p* < 0.01; multiple comparisons, uIgG-800F vs. uFab2-800F *p* < 0.01, uIgG-800F vs. uFab-800F *p* < 0.01, uFab2-800F vs. uFab-800F *p* < 0.01). MFI of the other organs did not approach or pass that of tumor MFI’s and did not significantly impact background fluorescence. Ex vivo macroscopic tumor fluorescence could be clearly visualized with all three tracers. uIgG-800F tumor MFI, however, was consistently higher than uFab2-800F or uFab-800F in all three orthotopic tumor models (Fig. 6b). *Post-mortem* overlay of histology with fluorescent signal confirmed tumor-specific accumulation at the tumor cells using all three tracers (Fig. 6c).

## Discussion

Optimizing NIR contrast agents for molecular imaging is a challenge, as tumor visualization is dependent on a multitude of factors including, but not limited to, tracer size, valency, affinity, and labeling ratio [29–31]. By decreasing size, the current study evaluated whether tumor-specific imaging could be retained and concurrently pharmacokinetics improved for the validated humanized anti-uPAR monoclonal antibody MNPR-101. Although the 100-kDa F(ab’)_2_ and 50-kDa Fab performed similarly *in vitro* to the 150 kDa IgG, *in vivo* imaging resulted in more rapid FGS with the two smaller fragments at the cost of peak tumor fluorescence.

Only few reports have directly compared antibodies with their fragments for FGS. Fluorescent anti-HER3 IgG and single-chain variable fragment (scFv)-Fc had higher tumor MFIs compared to scFv, Fab, diabody, and scFv-C_H_3 in HNSCC xenografts [32]. In another study, EphB4-targeting IgG showed maximum tumor uptake while F(ab’)_2_ and Fab resulted in step-wise lower tumor signal [33]. In a third study, using ICAM-1 Fabs, tumor MFI decreased to such an extent compared to the full-sized antibody that it matched that of non-specific controls [34]. These results, although differing in constructs and/or targets, match ours and are comparable to previous PET studies where CD105 targeting F(ab’)_2_ and Fab lead to earlier imaging but lower peak signal [22, 23].

Previously, however, we achieved high peak tumor signal with the epithelial cell adhesion molecule (EpCAM) targeting Fab VB6-845-IRDye800CW resulting in clinical translation (NTR7570) [35]. Expression characteristics of the targeted receptor could explain this difference in peak MFI attained by the Fabs. The colorectal HT-29 and breast MCF-7 cell lines used by Boogerd et al. express respectively 195,000 and 260,000 copies of EpCAM while BxPc-3, OSC-19, and HT-29 have 10,000–30,000 copies of uPAR [14, 35]. For receptors with lower cell expression, like uPAR, HER3, and EphB4, longer circulation times could be required to establish sufficient accumulation of tracer in tumors and clearance from background tissue [32, 36, 37].

In addition to receptor expression, tracer extravasation and clearance can clarify differences in tumor uptake. IgGs compensate their unfavorable slow extravasation characteristics with extended circulation times, in part due to their Fc receptor-mediated recycling and minimal renal clearance, providing a tracer multiple chances for extravasation [17, 38]. In contrast, the improved extravasation of molecules ranging in size between 20 and 50 kDa is not in proportion to the rapid renal clearance resulting in worsened tumor accumulation compared to IgGs [39, 40]. At and below 20 kDa, like for nanobodies and peptides, renal clearance is practically at first pass and as such cannot improve, while extravasation increases rapidly with decreasing size resulting in favorable tumor accumulation again [40]. Once in the tumor compartment, the superior diffusion of smaller molecules will result in a more homogenous tumor distribution compared to their larger counterparts [31].

Nonetheless, these disadvantages can be compensated by improving target affinity as there is an inverse relation between size and affinity (K_D_) required to reach similar tumor uptakes. K_D_s of 10^−8^ to 10^−6^ M for antibodies result in similar degrees of tumor retention as Fabs or scFvs with a K_D_ of 10^−10^ to 10^−8^ M [41–43]. Our anti-uPAR Fab has a nanomolar affinity, approximately ten-fold higher than the ICAM-1 Fab, and similar to the HER-3 Fab while VB6-845-IRDye800CW has a picomolar affinity [35]. In conclusion, the combination of receptor density and tracer affinity in combination with size plays a pivotal role in achieving high peak intensity, possibly explaining why VB6-845-IRDye800CW achieved superior imaging conditions and why, for the other targets, the larger agents resulted in superior MFIs.

The currently described uPAR-targeting antibody fragments are not the only uPAR-targeting contrast agents under development. The growth factor domain of urokinase (ATF), the natural ligand of uPAR with a K_D_ of 2.5 × 10^−10^ M, has been coupled with various dyes like Cy5.5 and NIR830 for FGS as approximately 18-kDa peptides and much larger nanoparticle probes [44–46]. The 9-mer peptide ICG-Glu-Glu-AE105 also targets the uPAR-ATF interaction with a K_D_ of 134 × 10^−9^ M and results in rapid (6–24 h) tumor localization in various *in vivo* xenograft models [47–49].While the delayed imaging window of uIgG-800F and rapid imaging window of ATF peptides and ICG-Glu-Glu-AE105 are evident, peak fluorescence is much harder to compare as these constructs not only differ in size and affinity but also in fluorophore. In this case, IRDye800CW has a higher extinction coefficient and quantum yield than ICG and can be expected to be brighter [50]. Lastly, the targeting epitope is a crucial difference between ATF peptides, ICG-Glu-Glu-AE105, and huIgG-800CW where the former two, as uPA competitors, are dependent on low endogenous uPA expression and the latter is not [51]. Co-injection experiments of uPA and ICG-Glu-Glu-AE105 have resulted in an almost 50% decrease in fluorescence signal [47]. huIgG-800CW specifically targets the D2-D3 uPAR isotype, often found to be overexpressed in cancer [9, 16]. 

While the conclusions drawn in this study reflect the literature, the current study contains a couple limitations. Although the limited group sizes were sufficient according to previous sample size calculations to identify the most relevant differences in TBRs (increase in 50%, see ESM), more subtle differences could have been missed. Ethical constraints, however, limited researching this avenue. In addition, using orthotopic models minimalized the time points tumors could be visualized *in vivo*, potentially missing better imaging moments. This was negated by first imaging the tracers serially in a subcutaneous tumor model and carefully defining what determined a suitable time window (see ESM). Lastly, administrating tracers based on fluorescence as opposed to antigen binding sites could potentially have allowed for a more accurate comparison of fluorescence intensity; however, the results, if different at all when injecting a surplus amount of tracer, would have skewed the conclusion even more towards full-sized antibodies.

## Conclusions

To conclude, this study successfully introduces two novel uPAR antibody-fragment tracers based on the extensively validated MNPR-101 humanized parental antibody. (F(ab’)_2_ and Fab greatly improved time-to-imaging while the whole antibody demonstrated superior peak fluorescence. In the clinic, the various pharmacokinetic profiles of the tracers should be considered as Fab utilization is better in (semi-) acute settings (same-day or next-day surgery), but should not be used when absolute receptor expression is expected to be relatively low. In these cases, surgeons should veer towards full-sized antibodies or constructs smaller than 20 kDa, such as nanobodies or peptides.

## Supplementary Information

Below is the link to the electronic supplementary material.

Supplementary file1 (DOCX 1354 KB)

Supplementary file2 (PDF 86 KB)
