# Supplementary material for: Side-by-Side Comparison of uPAR-Targeting Optical Imaging Antibodies and Antibody Fragments for Fluorescence-Guided Surgery of Solid Tumors
Source: Mol Imaging Biol. 2021 Oct 12;25(1):122–32. doi: 10.1007/s11307-021-01657-2 (PMC9970952; doi:10.1007/s11307-021-01657-2)
Supplement: Supplementary file 1 — Supplementary file1 (DOCX 1354 KB) [file 11307_2021_1657_MOESM1_ESM.docx]

Electronic Supplementary Material: ESM

*Surface plasmon resonance of antibody fragments*

All surface plasmon resonance (SPR) experiments were performed on a Biacore T200 (GE Healthcare Life Sciences, Uppsala, Sweden) using a NiHC1500M sensor chip (Xantec Bioanalytics, Düsseldorf, Germany) at 25°C. Before each run, the chip was equilibrated in HBS-P running buffer (10 mM HEPES, 150 mM NaCl, pH 7.4 with 0.05% Tween-20) with the addition of 2% BSA, at a flow speed of 30 µl/min. Channel 2 was used with channel 1 as reference channel lacking immobilized uPAR. A single-cycle kinetic method was used that included three assay steps. In step 1, residual nickel was stripped with a 300 s injection of 350 mM EDTA followed by equilibration in running buffer for 60 s. In step 2, a 5 mM nickel chloride solution was used to obtain a relative average immobilization level of 2000 resonance units (RUs), followed by the immobilization of uPAR, resulting in a relative immobilization level of up to 200 RUs. Step 3 involved a 600 s equilibration injection with running buffer followed by five injections of antibody fragment at increasing concentration with a contact time of 120 s, a stabilization time (in between injections) of 60 s, and a final dissociation time of 1200 s. Five serial dilutions were used for each antibody fragment. A kinetic analysis was performed using the Biacore T200 Evaluation software version 3.2 (GE Healthcare Life Sciences, Uppsala, Sweden) using a single-cycle kinetic 1:1 binding model yielding association, dissociation, and affinity constants.

*Cells and culture conditions*

HEK empty vector (HEK EV), HEK uPAR wildtype (HEK uPAR WT) and HEK uPAR D2-3 isoform (HEK uPAR D2-3) [15] were cultured in Dulbecco’s modified eagle medium (DMEM; 61965-026 Gibco, Life Technologies, California, USA) supplemented with 10% fetal bovine serum (FBS; F7524, Sigma-Aldrich, Missouri, USA), 1% penicillin-streptomycin (PS; 09-757F, Lonza, Basel, Switzerland) and 0,5 mg/ml G418 (10131-19, Gibco, Life Technologies, California, USA). For orthotopic mouse models cells transfected with luciferase 2 (luc2) were used to follow tumor growth by bioluminescence imaging (BLI). OSC-19-luc2-GFP cells were cultured in DMEM supplemented with 10% FBS and 1% PS. BxPC-3-luc2 and HT29-luc2 were cultured in RPMI-1640 (21875-034, Gibco, Life Technologies, California, USA) supplemented with 10% FBS and 1% PS. All cells were grown at 37°C and 5% CO_2_ in a humidified incubator and routinely screened for *Mycoplasma* infection by polymerase chain reaction.

*Flow cytometry*

80 - 90% confluent cells were detached with trypsin/EDTA. To allow adhesion molecule regeneration, cells suspended in culture media were incubated at 37°C for 3 hours on a rocker at 100 rpm. Cell viability was assessed with trypan blue followed by resuspension in ice-cold phosphate-buffered saline (PBS) at 500,000 cells/tube and washing. Cells were incubated with 5 µg/ml primary antibody (-fragment) for 30 minutes, washed thrice and incubated with 5 µg/ml anti-human secondary IgG Fab fragment antibody (clone 4A11; ab771, Abcam, Cambridge, UK). The wash cycle was repeated and cells were incubated with a tertiary anti-mouse 488 antibody for visualization (1/800; A21121, Life Technologies, California, USA). After washing and suspension in 1:4000 propidium-iodide:PBS, cells were measured on a LSRII flow cytometer (BD Biosciences, California, USA). Data was analyzed using FlowJo™ (BD, version 10.6.1).

*Cell-based plate assays*

50,000 cells per well in a 96-wells plate (3596, New Jersey, USA) were cultured until 80 – 90 % confluency. Plates were washed twice with ice-cold PBS supplemented with 0.5% bovine serum albumin (0.5% PBSA) and incubated with 0 – 20 µg/mL of 800F labeled antibodies for 60 minutes. Nonbinding antibodies were washed away and fluorescence intensity was measured using the 800 nm channel of the Odyssey imager. Cells were permeabilized with 40/60 % acetone/methanol, washed, and incubated with the nucleic acid stain ToPro-3 iodide (T3605, Invitrogen, California, USA) for 5 minutes. After another washing cycle, nucleus fluorescence was measured using the 700 nm channel of the Odyssey to correct 800 nm signal for cell density. Experiments were performed in triplet and repeated trice.

In vivo *experimental set-up*

This study included three experimental groups (1 nmol uIgG-800F, 1 nmol uFab2-800F and 2 nmol uFab-800F) in four different models (subcutaneous, PDAC, H&N and CRC PC orthotopic models). Experimental groups consisted of 3 mice per group where each mice was considered a separate experimental unit (total mice = 36) . Sample size was based on previous sample-sized calculations for similar experiments. Briefly, use the same-size calculator G*Power (Heinrich Heine Universität Düsseldorf) and a reference TBR of 2.11 ± 0.44 and a relevant difference defined as an increase or decrease of 50%, the minimum group size was defined as n = 3. Mice were randomly allocated to an experimental group once tumors reached predetermined cut-off sizes using an online randomizer to minimize potential confounders ( > 50 mm^3^ for subcutaneous tumors and > 1.0x10^8^ p/sec/cm2/sr for orthotopic models). Mice that did not develop tumors were excluded. Researchers were not blinded. The primary outcome measurement was TBR. Secondary outcome measurements were MFI and Artemis exposure time.

*Determination of optimal imaging-window*

Optimal imaging windows were selected based on a combination of characteristics. First of all the TBR had to be larger than 2 (PMID 28874886). Secondly the MFI had to be suitable for real-time imaging using the Artemis clinical camera (defined here as fluorescence of 0.1 a.u. as measured with the Pearl Imager). These two variables gave a range within which tumors could be imaged clearly and the imaging time was subsequently set using our experience with similar tracers, the literature and visual interpretation of the serial imaging.

Supplementary Table 1: K_a_ and K_d_ values of huIgG and its fragments

| Tracer | K_a_ (1/Ms) | K_d_ (1/s) |
| --- | --- | --- |
| uIgG | 3.57x10^5^ | 7.78x10^-5^ |
| uIgG-800F | 6.05x10^5^ | 1.27x10^-4^ |
| uFAb2 | 5.47x10^5^ | 2.92x10^-4^ |
| uFab2-800F | 4.47x10^5^ | 2.46x10^-4^ |
| uFab | 4.00x10^5^ | 4.08x10^-4^ |
| uFab-800F | 1.94x10^5^ | 3.08x10^-4^ |


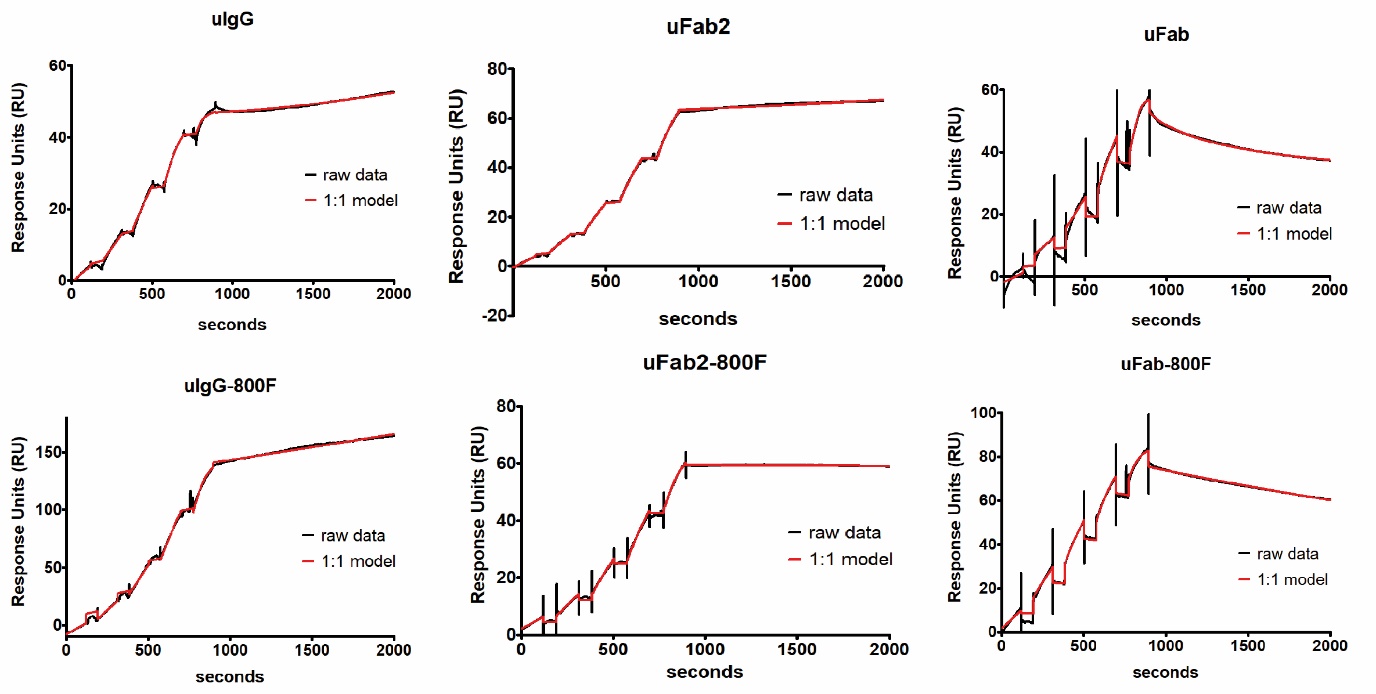


Suppl. Fig 1. SPR sensograms of unconjugated and conjugated uIgG, uFab2 and uFab.


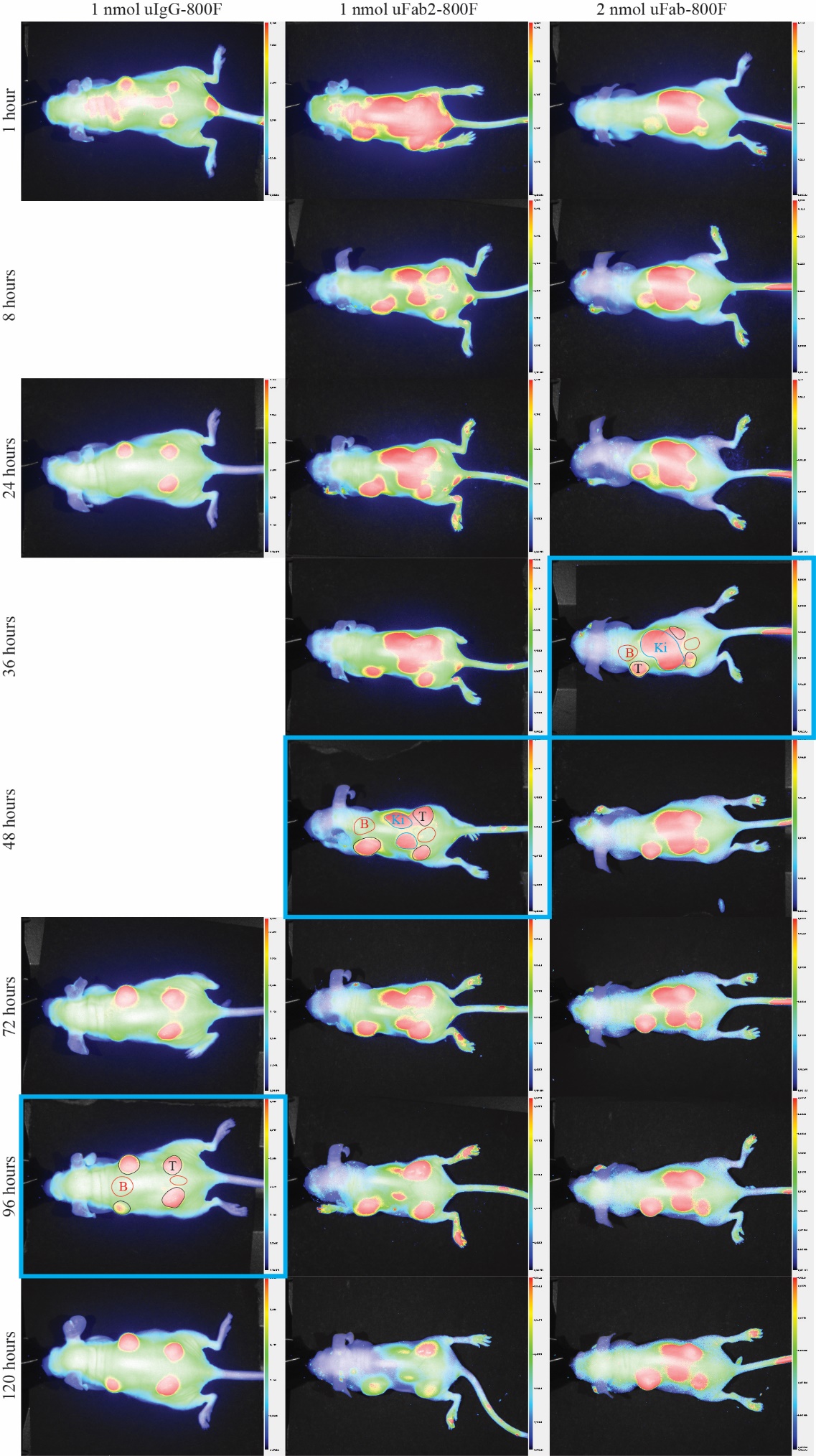


Suppl. Fig. 2 Serial fluorescence imaging of subcutaneous tumors: after intravenous injection of 1 nmol uIgG-800F, 1 nmaol uFab2-800F and 2 nmol uFab-800F subcutaneous HT-29 tumor-bearing mice were serially imaged for a period of 5 days with the Pearl. In representative images tumor (T), kidney (Ki), background (B) fluorescent regions-of-interest are outlined in black, blue and red, respectively.


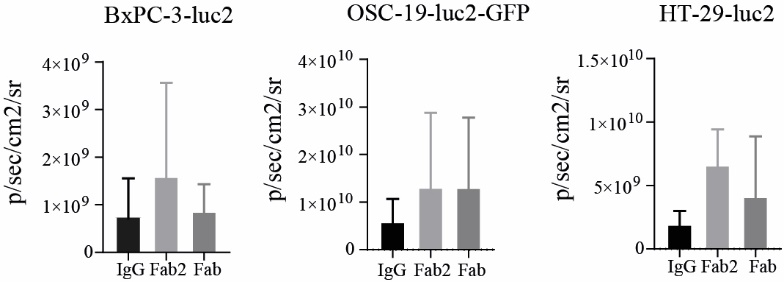


Suppl. Fig. 3 Bioluminescence signal of tumors at time point of intravenous injection of the fluorescence tracer of BxPC-3-luc2, OSC-19-luc2-GFP, and HT-29-luc2 tumors.
